# Paediatric pancreatic trauma in North Queensland: a 10-year retrospective review

**DOI:** 10.1186/s12887-023-03904-7

**Published:** 2023-02-21

**Authors:** Emily Everson, Helen Buschel, James Carroll, Pranavan Palamuthusingam

**Affiliations:** 1 Townsville University Hospital, 100 Angus Smith Drive, Douglas, Townsville, QLD 4810 Australia; 2 James Cook University, 1 James Cook Drive, Townsville, QLD 4810 Australia

**Keywords:** Pancreas, Pediatrics, Trauma, Abdominal injuries

## Abstract

**Purpose:**

To establish the incidence of pancreatic trauma in North Queensland to the region’s only tertiary paediatric referral centre, and to determine the patient’s outcomes based on their management.

**Methods:**

A single centre, retrospective cohort study of patients < 18 years with pancreatic trauma from 2009 to 2020 was performed. There were no exclusion criteria.

**Results:**

Between 2009 and 2020 there were 145 intra-abdominal trauma cases, 37% from motor vehicle accidents (MVA), 18.6% motorbike or quadbike, and 12.4% bicycle or scooter accidents. There were 19 cases of pancreatic trauma (13%), all from blunt trauma and with associated injuries. There were 5 AAST grade I, 3 grade II, 3 grade III, 3 grade IV injuries, and 4 with traumatic pancreatitis. Twelve patients were managed conservatively, 2 were managed operatively for another reason, and 5 were managed operatively for the pancreatic injury. Only 1 patient with a high grade AAST injury was successfully managed non-operatively. Complications included pancreatic pseudocyst (*n* = 4/19; 3 post-op), pancreatitis (*n* = 2/19; 1 post op), and post-operative pancreatic fistula (POPF) (*n* = 1/19).

**Conclusion:**

Due to North Queensland’s geography, diagnosis and management of traumatic pancreatic injury is often delayed. Pancreatic injuries requiring surgery are at high risk for complications, prolonged length of stay, and further interventions.

## Introduction

Our institution is a regional tertiary trauma and paediatric referral centre for North Queensland, serving an area of 780,000 km^2^. Smaller hospitals across the region may manage simple paediatric cases, often in liaison, but serious paediatric trauma, particularly pancreatic trauma, are transferred.

Pancreatic trauma in children, although uncommon, is a serious condition associated with high morbidity [1, 2]. Blunt pancreatic trauma is more common than penetrating injuries, though remains rare. The incidence of traumatic pancreatic injuries in children with blunt abdominal trauma is less than 10%, with the pancreas being the fourth most commonly injured solid organ after the spleen, liver, and kidneys [2]. The most frequent aetiologies include motor vehicle accidents, followed by domestic violence and bicycle accidents [3, 4]. Penetrating injuries, such as gunshot and stabbing injuries, are rare [1].

Pancreatic trauma is difficult to diagnose, with symptoms often insidious in onset and non-specific [4]. Abdominal symptoms including nausea or vomiting, and abdominal pain, do not correlate with trauma severity [1]. Serum amylase and lipase are elevated in the majority of patients with pancreatic trauma. However, they may be normal in the first few hours of trauma [1, 2]. Serial tests may thus be appropriate. Serum lipase is considered more specific for pancreatic injury, but neither test is diagnostic [2]. Positive results should prompt cross-sectional imaging [5]. However Computed Tomography (CT) of the abdomen has now become standard as part of the early initial workup of most paediatric trauma patient with a significant mechanism of injury.

Evaluating the integrity of the pancreatic duct is integral to decision-making. CT is the first-line investigation. It aids with pancreatic injury grading as well as assessment for other life-threatening injuries [6]. Direct and indirect signs of pancreatic duct injury on CT include complete gland transection, > 50% laceration through the gland, or the presence of early peripancreatic fluid [2]. Other imaging modalities may offer better visualisation of the duct. Magnetic resonance cholangiopancreatography (MRCP) is more sensitive than CT for identifying disruption of the pancreatic duct [7]. Endoscopic retrograde cholangiopancreatography (ERCP) likely offers the greatest specificity in defining duct injury, as well as offering therapeutic potential via pancreatic duct stenting. However, it is invasive and incurs the risk of post-procedural pancreatitis [2]. Its utility in children is unknown [2].

Definitive management of pancreatic trauma is generally governed by injury grade, as determined by cross-sectional imaging (see Table 1). Ductal injuries (Grade 3 and above), are considered high-grade and have high likelihood of pancreatic leak if not addressed surgically. Resection is usually indicated for distal injuries. Proximal to the neck of the pancreas, resection is challenging, and external or endoscopic drainage is recommended. These patients often have complex associated injuries, including the duodenum, and are especially challenging. These recommendations are based on adult trauma, however, and no specific paediatric pancreatic trauma guidelines exist.

**Table 1:** AAST organ injury scale for pancreatic injuries [5]

Grade	Type of Injury	Description of Injury
I	Haematoma	Minor contusion without duct injury
	Laceration	Superficial laceration without duct injury
II	Haematoma	Major contusion without duct injury or tissue loss
	Laceration	Major laceration without duct injury or tissue loss
III	Laceration	Distal transection or parenchymal injury with duct injury
IV	Laceration	Proximal transection or parenchymal injury involving ampulla
V	Laceration	Massive disruption of pancreatic head

The conservative management of solid-organ trauma was pioneered in paediatric trauma. In the absence of clear evidence-based guidelines, it is presently unknown if the future of pancreatic trauma management lies in a similar non-operative direction [8]. Whilst it is fairly clear that low-grade pancreatic injuries can be managed safely without surgery, the natural history of high-grade pancreatic trauma lends itself less well [1, 2, 4]. Non-operative management involves close monitoring of the patient’s condition, consideration to repeated bloods and imaging, with or without total parenteral nutrition (TPN) [5]. Surgery may be associated with shorter length of stay, reduced complications, and potentially reduced morbidity [2, 9]. Non-operative management may reduce early surgical complications and mortality, but opens the door to later pancreatic complications [2, 9]. These include pancreatic pseudocyst formation, peripancreatic fluid collections, pancreatitis, and pancreatic fistula [1, 10].

This study aimed to assess the epidemiology of pancreatic trauma and the diagnostic work up for children in North Queensland. Secondly, it aimed to provide current insight into the management of pancreatic trauma, especially the efficacy of non-operative management.

## Methods

This is a retrospective cohort study conducted at a regional tertiary hospital. The Townsville Hospital and Health Service Human Research Ethics Committee granted approval of this low risk research project, HREC/QTHS/65012 [11].

Paediatric patients were identified via clinical coding, with inclusion of all patients less than 18 years of age coded as ‘injury intra-abdominal organs’ (S36) as the principal or associated diagnosis, between January 2009 and September 2020. Data was extracted from the local health electronic medical record and local pathology provider database. This yielded a total of 145 intra-abdominal organ traumas. Patients without pancreatic trauma were then excluded following review of the 145 patient charts, leaving 19 patients meeting inclusion criteria. Pancreatic injury was defined by the presence of injury on CT. An elevated lipase > 3 times the normal limit without CT findings of pancreatic trauma was defined as traumatic pancreatitis.

Data was collected regarding patient demographics, Aboriginal and Torres Strait Islander status, location of residence, mechanism of injury, clinical presentation, laboratory findings, and imaging findings. Outcomes recorded included paediatric intensive care unit (PICU) admissions, need for TPN or nasogastric (NG) feeds, endoscopic intervention, operative management and operative findings, length of hospital stay, and pancreatic complications.

Data obtained from eligible patients was entered into an electronic spread-sheet, and statistical analysis was performed using SPSS.

## Results

**Fig. 1:**
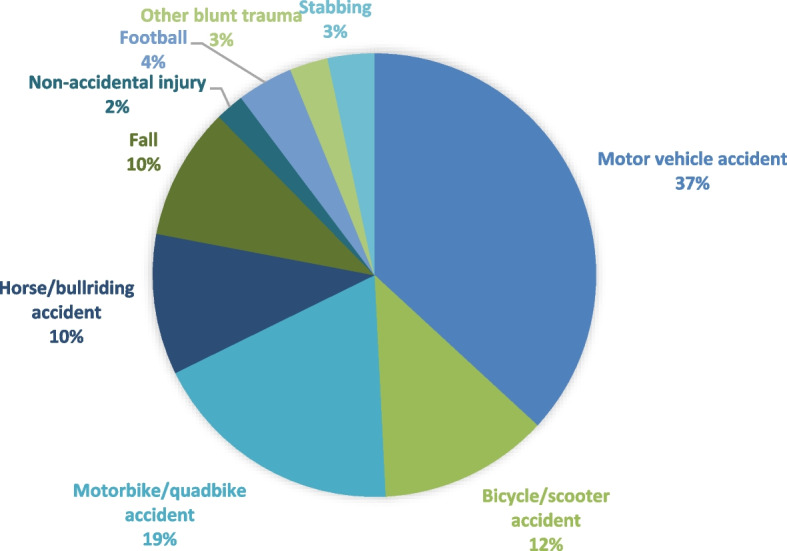
Mechanisms of intra-abdominal trauma

From 2009 to 2020 there were 145 intra-abdominal trauma cases, aged 7 months to 17 years (mean age 11.42 yrs). Of these cases, 73% (*n* = 106) were male and 27% (*n* = 39) were female. The most prevalent age group was between 12 and 15. Indigenous children were overrepresented, accounting for 25.5% (*n* = 37) of cases compared to 10% of the population. Of all intra-abdominal trauma, 37% (*n* = 53) was caused by motor vehicle accidents, 12.4% (*n* = 18) bicycle or scooter accidents, 18.6% (*n* = 27) motorbike or quadbike accidents, and 10.3% (*n* = 15) horse or bull-riding accidents. Additional mechanisms of trauma are detailed in Fig. 1.

**Fig. 2:**
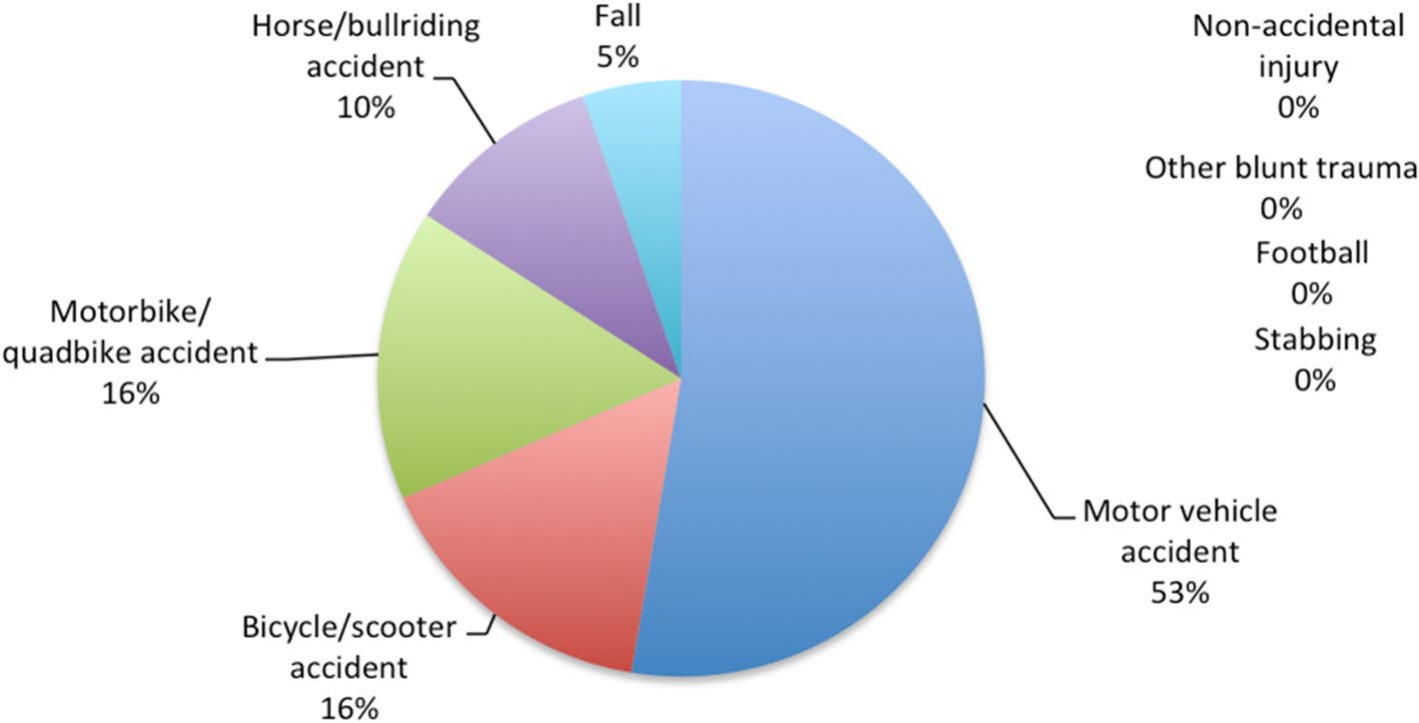
Mechanisms of pancreatic trauma

Pancreatic trauma occurred in 13% (*n* = 19) of the 145 intra-abdominal trauma cases. Indigenous children were again overrepresented, accounting for 36.8%. Pancreatic trauma occurred in patients aged 1 year to 17 years (mean age 13 years). All 19 cases were from blunt trauma, most commonly being a consequence of a MVA (53%, *n* = 10). Other causes included bicycle or scooter accidents (*n* = 3), motorbike or quadbike accidents (*n* = 3), horse or bull-riding accidents (*n* = 2), and falls (*n* = 1). As shown when comparing Figs. 1 and 2, MVA and Bicycle/Scooter Accidents were more common mechanisms of injury for pancreatic trauma compared to all intra-abdominal trauma. Over half of patients were transferred from rural or non-tertiary regional hospitals (*n* = 12). All cases of pancreatic trauma had associated injuries, with 12 having associated intra-abdominal injuries, as detailed in Table 2.

**Table 2:** Summary of pancreatic trauma (*n* = 19)

Injury	Initial Lipase	AAST Grade	Management	Additional abdominal injuries	Complication/s	LOS
Traumatic pancreatitis	1410	0	Conservative	Nil	Nil	5
Traumatic pancreatitis	922	0	Conservative	Nil	Nil	7
Traumatic pancreatitis	132	0	Conservative	Nil	Nil	2
Traumatic pancreatitis	337	0	Conservative	Nil	Nil	2
Pancreatic contusion	32	1	Conservative	Nil	Nil	10
Pancreatic contusion	33	1	Conservative	Spleen	Nil	17
Pancreatic contusion	34	1	Trauma laparotomy for bowel injury, pancreas left alone	Bowel	Nil	15
Pancreatic contusion	23	1	Conservative	Spleen, kidney	Nil	12
Pancreatic tail laceration	38	1	Conservative	Liver, kidney	Nil	4
Pancreatic tail laceration	403	2	Conservative	Liver, spleen	Nil	10
Pancreatic neck laceration	21	2	Delayed laparotomy for small bowel injury	Spleen, small bowel	Nil	10
Pancreatic body laceration	478	2	Delayed laparotomy for pseudocyst	Spleen	Pseudocyst (pre-op)	57
Pancreatic body transection	1410	3	Trauma laparotomy for spleen and kidney, pancreas left alone	Spleen, kidney	Pseudocyst	25
Pancreatic body transection	137	3	Spleen-preserving distal pancreatectomy	Nil	Nil	12
Pancreatic body transection	680	3	Spleen-preserving distal pancreatectomy	Nil	Nil	12
Pancreatic neck transection	3480	4	Conservative	Liver, kidney	Pancreatitis	35
Pancreatic neck transection	113	4	Laparotomy and evacuation hematoma	Liver	Pseudocyst	66
Pancreatic neck transection	1130	4	Delayed surgery for duodenal injury. Pseudocyst ultimately required pancreaticojejunostomy	Liver, spleen, kidney, duodenum	Pseudocyst, POPF, chronic pancreatitis	148
Pancreatic transection at body/neck region	406	Unknown (grade 3 or 4)	Laparotomy and thoracotomy. Pancreatic debridement, ends oversewn and sealed with tisseal	Liver, kidney, portal vein, duodenum, ruptured diaphragm	Wound infection	36

Of the 19 patients, 14 had abdominal pain. Other symptoms or signs were nausea and vomiting (*n* = 8, 42%), abdominal bruising (*n* = 6, 32%), and back pain (*n* = 4, 21%). Observations included tachycardia for age (*n* = 6, 32%), tachypnoea for age (*n* = 3, 16%), hypotension for age (*n* = 3, 16%), hypertension for age (*n* = 1, 5%), and fever (*n* = 2, 11%). Initial pathology most frequently showed neutrophilia (*n* = 15, 79%), followed by liver function derangement (*n* = 14, 74%), leucocytosis for age (*n* = 11, 58%), and raised bilirubin (*n* = 4, 21%). The initial lipase level, which is from the patient’s first contact with any health service, was deranged in 14/19 patients (74%), with a median initial lipase level of 337 (range 21 to 3480). Four patients had elevated lipase in the setting of abdominal trauma but no CT evidence of contusion or laceration, and thus have been grouped as traumatic pancreatitis. Lipase was normal in all (3) cases of pancreatic contusion, and generally elevated otherwise. The lipase level did not correlate with grade of injury (Pearson Correlation 0.31).

Of the 19 patients, all 15 with American Association for the Surgery of Trauma (AAST) Grade I-IV pancreatic injuries had the injury diagnosed on CT. This occurred within 6 hours for 7 patients, within 24 hours for an additional 5 patients. Delayed imaging past 24 hrs occurred in three patients, all of whom were transferred from rural centres. Three patients also had MRCP, and 1 patient also had an ultrasound. PICU admission was required for 13 patients (68%). Eight patients required TPN, including all 7 patients managed operatively for the pancreatic injury. Length of stay (LOS) was longer following abdominal surgery (mean LOS 46.5, median 30, range 10–148), compared to non-operative management (mean LOS 10.4, median 9, range 2–35).

Of the 19 patients, 10 were managed totally non-operatively. Two underwent laparotomy without any management of the pancreas for low-grade injuries. Only one of 7 major pancreatic trauma patients was successfully managed non-operatively. One patient had late non-operative failure, requiring roux-en-y cyst-jejunostomy for pseudocyst.

There were two patients with isolated pancreatic trauma (distal lacerations) both managed with uncomplicated distal pancreatectomy. Four patients required surgery acutely, either at presentation or shortly after; these were all multivisceral injuries, including duodenal injury in two. Pancreatic drainage without formal resection was employed in these four cases, and three of these developed pseudocyst.

Complications included pancreatic pseudocyst (*n* = 4/19; 3 post-op), pancreatitis (*n* = 2/19; 1 post op), post-operative pancreatic fistula (POPF) (*n* = 1/19), and post-operative wound infection (*n* = 1/19). The mortality rate was 0%.

Please refer to Table 2 for further details regarding the 19 pancreatic trauma patients.

## Discussion

Our data suggested a 13% pancreatic injury rate in abdominal trauma, in line with recent meta-analysis (incidence < 10% annually) [2]. Notably, there was an overrepresentation of Indigenous patients, suggesting that these children are at increased risk of severe trauma. Current demographic data suggests that pancreatic trauma is very often associated with other injuries, which is consistent with the findings of this study [2]. Mortality rates have been quoted as approximately 5%, in this small population of pancreatic trauma there were no deaths [9, 12]. There was clear male predominance with male patients accounting for 73% of abdominal trauma cases, a finding consistent with existing demographic data [9].

An important consideration from the adult literature is that outcome from pancreatic injury is worsened by a delay in diagnosis [13]. This is pertinent to the outcomes of the children in this study due to the geographical challenges of healthcare in North Queensland. The Townsville Hospital paediatric surgery team manages patients from south of Mackay to the Torres Strait Islands, an area of 780,000km^2^. Fifteen of the 19 patients with pancreatic injuries were inter-hospital transfers from other regional, rural or remote areas. Of the 7 patients managed operatively, 6 were transferred from an outlying facility. It has been shown that when treatment is delayed, higher-grade pancreatic injuries with duct involvement have increased associated morbidity, mortality, and there is increased risk of deterioration. In these cases, the literature supports pancreatic resection where possible [8]. Additionally, it has also been shown that endoscopy and interventional radiology can improve the success of non-operative management [5]. ERCP is not available in children at our institution. The contributory delay to definitive diagnosis and management, and the lack of access to these non-operative management interventions, may have led to a lower threshold for operative intervention on arrival and a higher complication rate. Furthermore it could be argued these children had already trialled conservative management in some instances, and were transferred to Townsville Hospital with surgical intent.

These issues may contribute to the mean LOS of 26.7 days. A 2017 systematic review found an overall mean LOS of 21.7 days [9]. A 2021 meta-analysis showed shorter length of stay in the non-operative management group, although not significantly, coinciding with the trend observed in this study [2]. Higher-grade injuries would have logically a longer LOS due to more complex surgery and associated injuries, with complications more likely, making it difficult to compare the LOS between groups. Despite this, data also suggests that patients with failed non-operative management who subsequently have surgical intervention have the longest LOS [2].

Pancreatic trauma in children remains a diagnostic dilemma. This study also demonstrates the inconsistencies of the presenting symptoms and signs. Lipase was useful, being elevated in all major pancreatic trauma and all bar one lacerations. Lipase elevation did not occur in any patient with pancreatic contusion. CT gave the diagnosis in all cases, though follow-up imaging was performed in several patients. The pancreatic duct proved difficult to visualise on CT in children. For this reason, a laceration involving over 50% of the depth of the pancreas on CT is often taken as evidence of ductal injury [7].

ERCP offers highly accurate diagnosis of paediatric ductal injuries, and can also facilitate stent placement [2]. Whilst MRCP allows for better visualisation of the pancreatic parenchyma and is more sensitive for secondary signs of injury, it may not be superior for confirmation of duct integrity so may not be necessary [7]. If there is discrepancy between CT imaging and clinical findings, ERCP remains the gold standard to confirm duct disruption when considering pancreatic resection in children [7]. This is consistent with the current WSES-AAST Guidelines, recommending ERCP for both diagnosis and treatment even in the early phase after trauma in patients who are haemodynamically stable or stabilised [5]. It is unknown whether ERCP use would have reduced the need for surgical input. However, given the multivisceral nature of these injuries many would have required surgery for other reasons.

Recently, several authors have advocated earlier surgical intervention for cases of ductal injury [12]. Currently the success of non-operative management is quoted as 96% for grade I or II injuries, and 89% for grade III, IV, or V injuries [9]. In our study conservative management of major pancreatic trauma proved unfeasible in the majority of cases; in many cases this appeared to be a consequence of the associated visceral trauma.

There are only three studies looking at paediatric pancreatic trauma that have been published in Australia [3, 13, 14]. Jacombs et al. had a cohort of 65 children with pancreatic injuries, all were blunt injuries, and most were low grade. This study did not make a conclusion regarding conservative versus operative management [3]. The second study concluded non-operative management in the absence of complete duct transection was safe [14]. Sutherland et al. concluded that most children can be treated conservatively, with surgical intervention being limited to high-grade ductal injury [13]. All studies reported high rates of other intra-abdominal injuries, consistent with this study. The literature would suggest benefit in an early surgical approach for patients with major pancreatic trauma with ductal injury [2]. Certainly in our experience, distal injuries managed with resection did well. This highlights the need to definitively diagnose duct injury as soon as possible, so informed treatment decisions are made.

Our study serves as a highlight to the management of these injuries in the regional setting, with delayed diagnosis and transfer not uncommon. As the only local paediatric surgery centre, it is likely to have captured the majority of pancreatic trauma amidst this population. Limitations of this study include the small sample size, the retrospective nature allowing for bias from unmeasured confounders, and lack of longitudinal follow-up.

Overall, what can be concluded from this study and current literature is the history of MVA or handlebar injury should always prompt the attending clinician to consider pancreatic trauma. Clinical findings in children are often nonspecific, so diagnosis will require imaging. This should be done in conjunction with the paediatric surgical team, as the decision to perform cross-sectional imaging on children is often difficult. Early transfer should be considered, particularly in Australia where large distances may delay this.

The decision to operate is complex and depends on the location of the injury, the grade, associated intra-abdominal injuries and the physiological status of the patient. Distal injuries are more amenable to resection, but the benefit of this over a conservative approach is unknown. The role of ERCP, in both diagnosis and therapeutic stent placement, remains unclear. Where surgery is performed, particularly in the setting of failed conservative therapy, these patients can expect prolonged inpatient stay, and appropriate counselling should be performed.

Further studies are needed to establish definitive guidelines, to determine the risks and benefits of endoscopy and interventional radiology in AAST grade III-V pancreatic injuries, and to determine if there are some settings where patients with ductal injury can be managed non-operatively [9].

## Data Availability

The data that support the findings of this study are available on request from the corresponding author EE. The data are not publicly available due them containing information that could compromise research participant privacy.
